# Extracts and Abstracts

**Published:** 1888-11

**Authors:** 


### EXTRACTS AND ABSTRACTS.

### THE MODERN MEDICAL CURRICULUM.

Though the regulations made by the General Medical Council for the conduct of medical education in the United Kingdom are simple in principle, yet their practical application by the numerous universities and corporations involves a good deal of complexity. The council lays down a minimum curriculum and a minimum standard of knowledge, and no student can be admitted into the profession who does not attain to this standard. Broadly speaking, the regulations amount to the following: (1) The would-be student must pass an examination in general knowledge. A long list of the examinations which are considered adequate will be found on the first page of this number of the *Journal* (September 8, 1888). Having passed this test, the student can enter his name on the *Students' Register* kept by the General Medical Council. He is next required (2) to pass through a course of instruction in the preliminary sciences, chemistry, and biology, and to pass an examination therein. (3) He next receives instruction in anatomy and physiology—that is to say, the structure and functions of the body in health, and is examined in these subjects. Finally, the concluding part of the obligatory curriculum is given to (4) the study of diseased processes; and the student must pass examinations in medicine, surgery, and midwifery before obtaining a degree or diploma entitling him to practice.

The medical courses of all the universities and corporations run on parallel lines, and the familiar simile is peculiarly applicable in this instance, for as two parallel lines can never meet however far they may be prolonged, so the curricula of the universities and corporations never intersect, and the student once committed to a particular line finds it always difficult and often impossible to reach another, and as it may then appear to him more desirable, line without retracing his steps nearly to the beginning. An initial mistake, therefore, is liable to entail very serious consequences. The possession of the degree of M.D. is rightly regarded by all practicing medical men in this country as a distinct advantage, yet a very large proportion find themselves debarred from obtaining it, because the Conjoint Boards of England, Ireland, and Scotland, formed by the union of the medical corporations in London, in Dublin, and in Edinburgh and Glasgow, respectively, have no power to grant degrees. A Royal Commission is now sitting to inquire into the needs of the higher education in London, and it will in all probability recommend such reforms as will place degrees in medicine within the reach of all diligent medical students in London. Meanwhile, remembering the uncertainty of legislation, and the dilatoriness of government offices, it is the part of prudence to advise every young man at the outset of his career to consider well whether the degree of one of the existing universities be not within his means. The facilities for obtaining such degrees have notably increased within the last decade. The medical school of Cambridge, for instance, has grown to be one of the largest and most important in the country; the teaching, organized with the greatest care, is carried on by professors of the highest eminence, who have the command of excellent laboratories, and all the most approved educational appliances. While this school is taking its proper place in the life of its university, the medical teaching of the sister university has also been brought into a state of great efficiency, and the course of study for the degree of M.B.Oxon. has been carefully arranged to meet the needs of the modern student. Even the University of London, which has so long maintained an obstructive, if not retrograde, attitude, has promised certain important reforms which will make it possible to obtain its degrees at a less sacrifice of time and of opportunities for gaining practical knowledge. The official publication of these alterations can not be much longer delayed. Whatever may be the outcome of the present agitation a metropolitan student who has passed the earlier examinations of the university will find himself at the least in as good a position and not improbably in a better than those who may wait in the hope that "something may turn up." The creation of the Victoria University and the affiliation with it of the Owens College in Manchester, the Yorkshire College in Leeds, and the University College in Liverpool is another of these changes of recent years which have brought medical degrees within the reach of a much larger proportion of students; those who enter at one of these medical schools with a view to the degree of the Victoria University will not regret their choice. The University of Durham, with which the medical school of Newcastle-on-Tyne is affiliated, offers opportunities for gaining a degree in medicine of which students have been, perhaps, too slow to take advantage. There is, therefore, an extensive choice before the student, who will, moreover, find that all the English universities, with the exception of the Victoria University, allow a good deal of latitude in the choice of a school. Students at Oxford and Cambridge, for instance, after passing examinations in anatomy, physiology, and the preliminary sciences, can proceed to a metropolitan hospital, and take advantage of the unrivaled advantages for gaining clinical knowledge which London affords.

Of the four Scotch universities it is not necessary to speak here at length; the advantages which they offer to students are sufficiently evidenced by the large number who annually cross the Tweed in search of education and medical degrees in Scotland. Each university is a world sufficing for itself; between them there is no reciprocity, and their regulations are inelastic; Edinburgh, however, permits its undergraduates to pass limited periods of study in certain other schools. In Ireland, degrees in medicine are granted by both the universities, and the regulations of the Royal University will be found to have been drawn up in a liberal and practical spirit, in sympathy with the circumstances of modern life.

The choice of the student who is content to forego the possession of the degree of M.D., or to trust to the chapter of accidents to place it within his reach in the future, has been considerably limited in recent years by the formation of Conjoint Boards. In England he must choose between the Conjoint Board of the Royal College of Physicians and Surgeons and the Apothecaries' Society; the former will present him with the double diploma ("a triple qualification") of the two colleges, and hopes before long to be able to grant also the degree of M.D.; the latter gives him its license, which is also a "triple qualification." In Ireland there are at present two Conjoint Boards, and in Scotland one, each granting a "triple qualification."

This slight sketch may serve as a clue to the great body of information contained in the preceding pages, and in conclusion we would again impress upon intending students and their friends the paramount importance of looking far to the future before taking the first step; it is a source of regret that this is so, and there is reason to hope that before many years are passed important reforms may be achieved, but meanwhile we have to deal with rules and regulations as they are and not as they ought to be.—*British Medical Journal.*

---

### THE UNITED STATES CENSUS.

The laborious work of the United States Census, 1880, has reached its twelfth volume, and Part II of that volume is one of especial interest to those engaged in the promotion of preventive medicine and sanitary science. It deals with mortality and vital statistics, subjects which in this country are included in the ordinary reports of the Registrar-General, and which do not profess to come within the range of the staff who compile our census returns.

One especial feature of the volume under consideration consists in the division of the States into groups or regions, the physical characteristics of which are more or less distinct. Thus, there are twenty-one grand groups, each such group being made up of a number of State groups. The first four comprise the Atlantic and Gulf coasts, which primarily possess a sea climate where the extremes of heat and cold are mitigated by the presence of "that great balance-wheel of temperature," the ocean. Then come the several plateaus and hill districts, the region of the great northern lakes, the great river areas, the groups characterized by plains, heavily-timbered districts, the Cordilleran range, and lastly the Pacific coast group. Speaking generally of them, in relation to the influence of topographical features on special causes of death, it is shown that the conditions of climate, the amount of annual rainfall, the amount of low-lying and swampy land, age and sex, the distribution of the people, and the proportion of the colored and foreign population, appear to be the chief causes of the differences between the several grand groups, or between different portions of the same grand group. As regards the influence of geological formation it is held that, except in so far as it affects climate or drainage, the geology of the different regions does not appear to have exercised any marked influence upon the proportion of deaths from various causes, with the exception of goitre, urinary calculus, lead-poisoning, and, perhaps, such diseases of the digestive organs as are due to inorganic impurities in the water-supply. This experience coincides very much with that which has been obtained in this country.

The volume next deals at considerable length with a number of special diseases, and very carefully prepared maps are given, showing by different shades of color the different proportions which the number reported as to each of the several causes of death bears to the whole number reported; the State group being taken as the unit of area, except in the case of diphtheria, where the county is also taken as a unit. In some areas, such as the Indian Territory, no proper enumeration of deaths could be obtained; in others, such as New Mexico and Western Texas, the statistics could not be relied on as to some diseases; but in others, as in the Northern Mississippi group, it was found practicable to shade the maps so as to give an indication of the influence of great cities in the group. * *

Information of a similar character is supplied as regards a large number of other diseases and groups of diseases, and the statistics bearing upon the subject have been compiled in much detail and in a manner well calculated to serve the purposes of public health. The material embodied in this volume is in itself of the highest merit; but it will reach even a higher standard of value when in subsequent years detailed records extending over successive periods, and prepared with ever increasing accuracy, admit of being placed in comparison with each other.—*The Practitioner*.

Dr. John C. Peters says it would be an easy matter to clean city streets, and at the same time purify the air, by the issue of a dilution of bromine. Sixty cents worth can be diluted in three or four hundred gallons of water, which, if sprinkled on the streets, will be found effectual in purifying the germ-laden atmosphere of the heated city. Used on the streets it is one of the most powerful disinfectants. If city streets were sprinkled with a dilution of bromine, the sewers and the sub-ways flushed with it, and back alleys and the docks disinfected, it would do much to break up diseases which rage in certain districts of our cities.—*Herald of Health*.

### THE CANADIAN MEDICAL ASSOCIATION.

*The Canadian Practitioner*, for October, says, regarding the last annual meeting at Ottawa :

The recent meeting of this now well-established association was, in every respect, a very useful and pleasant one, and a success. The attendance was quite as large as usual.

The profession of Canada should support this association in the future more loyally than it has in the past. We would like to see three hundred instead of one hundred in attendance at each meeting. We want to keep up our connection with physicians in all parts of the Dominion, and the best way to do so is to meet at the annual gatherings of this, the most purely Canadian Medical Congress.

The association has now reached its majority. It came into existence in the same year as Confederation, and we think it can be fairly said that its success has been quite equal to that of our national progress. Many of the same difficulties which stand in the way of our national success are also barriers to the progress of our Dominion Medical Association. The chief among these is the distance between the different provinces, and the great disparity of interests, the result of such distances. It is probable, however, that in both cases the most troublesome times are past, and that the success of both will be more rapid and more steady in the future. * * *

The division of the association into three sections facilitated business very much. As was emphatically and truthfully stated by Dr. Mills, we, as members of the association, need to have greater energy and industry in preparing papers which will be of interest and profit to members of the profession generally.

Dr. H. P. Wright, of Ottawa, was elected president for the coming year.

### SEMOLINA IN DIABETES.

Mr. W. Stanley Armitage says: Some time ago I had the opportunity of watching a case of persistent glycosuria very closely, and found that at a time when the ingestion of a very small amount of toast, bread, or other farinaceous food was sufficient to produce Fehling's test afterwards in the urine, a large quantity of semolina pudding could be consumed without any such result. I was induced to try semolina after hearing of the method of its manufacture. As the quality of this food seems likely to vary, I should state that what was used in this case was obtained of an Edinburgh house, and was stated to be of the best. This I mention because, if carelessly prepared, no doubt much of the farinaceous element might be retained, though I am not aware that this ever is the case. Not having heard of semolina as a food in diabetes, I should much like to hear of any one who may have tried it, and also would request other medical men to see if it can be given in other and more pronounced cases of diabetes with a like good result. If such were the case, as it could be cooked in various forms, or baked as bread, it would add considerably to a class of food which is at present only far too limited.—*Lancet*, April 28, 1888.

